# Employment of digital gene expression profiling to identify potential pathogenic and therapeutic targets of fulminant hepatic failure

**DOI:** 10.1186/s12967-015-0380-9

**Published:** 2015-01-27

**Authors:** En-Qiang Chen, Lang Bai, Dao-Yin Gong, Hong Tang

**Affiliations:** Center of Infectious Diseases, West China Hospital of Sichuan University, No.37 Guo Xue Xiang, Wuhou District, Chengdu 610041 People’s Republic of China; Division of Infectious Diseases, State Key Laboratory of Biotherapy, Sichuan University, Chengdu, 610041 China; Institute of Basic Medicine, Chengdu University of Traditional Chinese Medicine, Chengdu, 610075 China

**Keywords:** Fulminant hepatic failure, Differentially expressed genes, Nuclear factor-kappa B, Therapeutic target

## Abstract

**Background:**

The dysregulated cytokine metabolism and activity are crucial to the development of fulminant hepatic failure (FHF), and many different cytokines have been identified. However, the precise gene expression profile and their interactions association with FHF are yet to be further elucidated.

**Methods:**

In this study, we detected the digital gene expression profile (DGEP) by high-throughput sequencing in normal and FHF mouse liver, and the candidate genes and potential targets for FHF therapy were verified. And the FHF mouse model was induced by D-Galactosamine (GalN)/lipopolysaccharide (LPS).

**Results:**

Totally 12727 genes were detected, and 3551 differentially expressed genes (DEGs) were obtained from RNA-seq data in FHF mouse liver. In FHF mouse liver, many of those DEGs were identified as differentially expressed in metabolic process, biosynthetic process, response to stimulus and response to stress, etc. Similarly, pathway enrichment analysis in FHF mouse liver showed that many significantly DEGs were also enriched in metabolic pathways, apoptosis, chemokine signaling pathways, etc. Considering the important role of nuclear factor-kappa B (NF-κB) in metabolic regulation and delicate balance between cell survival and death, several DEGs involved in NF-κB pathway were selected for experimental validation. As compared to normal control, NF-κBp65 and its inhibitory protein IκBα were both significantly increased, and NF-κB targeted genes including tumor necrosis factor α(TNFα), inducible nitric oxide synthase (iNOS), interleukin-1β, chemokines CCL3 and CCL4 were also increased in hepatic tissues of FHF. In addition, after NF-κB was successfully pre-blocked, there were significant alteration of hepatic pathological damage and mortality of FHF mouse model.

**Conclusions:**

This study provides the globe gene expression profile of FHF mouse liver, and demonstrates the possibility of NF-κB gene as a potential therapeutic target for FHF.

**Electronic supplementary material:**

The online version of this article (doi:10.1186/s12967-015-0380-9) contains supplementary material, which is available to authorized users.

## Introduction

Fulminant hepatic failure (FHF) is a severe, life-threatening disorder, which is characterized by a dramatic clinical syndrome resulting from massive hepatocyte death [1,2]. There is a large number of evidence showing that the dysregulated cytokine metabolism and activity are crucial to the progression of liver damage and the regulation of undesired inflammation is an attractive potential strategy in the treatment of FHF [3-5]. Though many different cytokines have been identified, the precise gene expression profile and their interactions in FHF are yet to be further elucidated.

In past decade, expression microarrays have been applied for genome-wide expression analysis for identification of differentially expressed genes (DEGs) associated with liver injury [6]. Although these studies provided new information regarding gene expression patterns in FHF, interactions between genes and affected pathways may have been missed because of technical limitations. Recently, by the rapid development of a “second generation” of sequencing technologies, the digital gene expression profile (DGEP) method was made [7-9], and millions of expression tags can be measured simultaneously at a fraction of the cost of capillary sequencing used in the original serial analysis of gene expression method. A major advantage of DGEP, which overcomes the drawbacks of using predesigned hybridization probes as in microarray-based methods, is that it allows detection of expressed genes in a strand-specific manner; and DGEP method provides information on the polarity of the expressed transcripts, allowing detection of sense and antisense transcripts from the same gene region.

At present, DGEP experiments often involve measuring the relative amount of mRNA expressed in two or more experimental conditions. This is because altered levels of a specific sequence of mRNA suggest a changed need for the protein coded for by the mRNA, perhaps indicating a homeostatic response or a pathological condition [10]. For example, if injured hepatic cells express higher levels of mRNA associated with a particular cytokine than normal cells do, it might be that this cytokine plays a role in hepatic injuries. Thus, the application of DGEP in transcriptome profiling of severe injured liver tissue is important to further understand the underlying mechanisms of FHF.

D-Galactosamine (GalN)/lipopolysaccharide (LPS)-induced liver failure in mice is dependent on macrophage-derived pro-inflammatory cytokines, including tumor necrosis factor (TNF)-α, interleukin (IL)-1β, IL-6 and interferon-γ, and accurately represents human FHF [11]. Thus, in this study, we detected global gene expression profile in normal and FHF mouse liver by DGEP method, and showed the main FHF-associated hepatic biological processes, pathways, and key molecules potentially serving as important therapeutic target for FHF.

## Materials and methods

### Animal experiments

Male BALB/c mice, 18–22 g, were obtained from Sichuan University (Chengdu, China). They were housed and cared for in rooms maintained at a constant temperature and humidity. Food and water were allowed ad libitum. Food was withdrawn the evening before the experiment. In this study, animal experiments involved in screening of DEGs in FHF mouse liver (Part. 1), and verification of some potential candidate genes related to the progression of FHF (Part. 2). In part. 1, a mouse model of FHF was established by intraperitoneal injections of D-GalN (800 mg/kg body weight; Sigma, Saint Louis, USA) and LPS (20 μg/kg body weight; Sigma), as described previously. Control mice were intraperitoneally injected with saline. Plasma and liver tissue samples were obtained 6 h after injection and were stored at -80°C until analysis. In part. 2, a potent NF-κB chemical inhibitor pyrrolidine dithiocarbamate (PDTC) was given by intraperitoneal injection to pre-block of NF-κB activation and expression, 12 h before D-GalN/LPS administration; and the plasma and liver tissue samples were also obtained 6 h after the construction of FHF mouse liver, and were stored at -80°C until analysis.

### Sample preparation and solexa library construction

Liver samples were ground in liquid nitrogen, and total RNA was extracted with Trizol reagent (Invitrogen). The integrity of total RNA was evaluated using an Agilent Bioanalyzer 2100. Solexa libraries were constructed following the manufacturer’s standard according to the schematic as described previously.

### Solexa sequencing and data analysis

The image files obtained from Illumina 1G sequencer were processed to produce sequence data. Then the high-quality reads were screened from the original raw data, and the adaptors were removed from each sequence. Subsequently, high quality clean tags were compared with RefSeq database and the expression level of each gene was normalized to transcripts per million (TPM). The significance of DGEP was analyzed as described previously. The expression pattern of these DEGs was visualized using the heat-map function in the R base package. In this study, to avoid the possible noise signal from high-throughput sequencing, the genes with an average TPM less than 1 were excluded; and the remained genes were used to calculate the fold changes and false discovery rate (FDR). And the absolute fold change no less than 1(|log_2_Ratio| ≥ 1) and FDR less than 0.001 were used to define the DEGs.

### Gene ontology functional and pathway enrichment analysis for DEGs

The clustered genes were assigned to molecular function, cellular component and biological process based on Gene Ontology using the web tool DAVID (http://david.abcc.ncifcrf.gov/home.jsp). Hypergeometric test was used to select the enriched molecular function, cellular component and biological process in Gene Ontology for each cluster. The pathways associated with these gene clusters were analyzed by KEGG (www.genome.jp/kegg/pathway.html).

### Detection of NF-κBp65 and its inhibitory protein IκBα in liver tissue

The protein expression levels of NF-κBp65 and its inhibitory protein IκBα in liver tissue were determined by western blot analysis, according to the standard manufacturer’s protocol. And the rabbit polyclonal anti-NF-κBp65 (Abcam, England) and anti-IκBα antibodies (Abcam, England) were both diluted at 1: 200; while rabbit polyclonal anti-GAPDH antibody (ProteinTech, USA) was diluted at 1:1000 as a control. Immunodetection was performed with the ECL-Plus kit (Pierce Biotechnology, USA), and immunoblot signals were quantified using Quantity One Software (Bio-Rad).

### Biochemistry and plasma protein analysis

In order to assess the extent of liver injury, serum alanine aminotransferase (ALT), aspertate aminotransferase (AST), total bilirubin (TBil) and indirect bilirubin (IBil) were measured with automatic analyzer (HITACHI, Japan). Serum levels of TNF-α were measured by enzyme-linked immunosorbent assay (GenStar BioSolutions, China), with lower limits of detection of 7 pg/mL.

### Determination of mRNA levels for candidate genes in liver tissue

Total RNA was extracted from frozen liver tissue with TRIzol Reagent (Invitrogen, USA). Total cellular RNA was reverse-transcribed using Moloney Murine Leukemia Virus (MMLV) reverse transcriptase (Gibco BRL, USA). Blank reactions with no RNA were performed in all experiments. The expression of candidate genes mRNA was measured in liver tissues by real-time PCR using Maxima SYBR green/ROX qPCR Master Mix (Fermentas Life Sciences, Canada). The primer sequences a of β-actin (the reference) and candidate genes obtained from DGE profiling experiment are listed in Table 1. And the fold changes of the expression of the candidate genes relative to the reference gene were calculated using the normalized expression (^Δ^Ct) method with default threshold values using CFX Manager Software (Bio-Rad).

**Table 1:** **Primer sequences used in the quantitative RT-PCR analysis**

**Gene**	**Length**	**Forward primers**	**Reverse primers**
TNFα	183 bp	CCCTCCAGAAAAGACACCATG	CACCCCGAAGTTCAGTAGACAG
TNFr	172 bp	GCAAAATCCCCCATACTCAAG	AGACCTATTTGGCACTCGCA
IκBα	180 bp	CCCTTACTGGAGAGACCCGA	GCAACAGAATAGCACCGACG
iNos	215 bp	GGGAATCTTGGAGCGAGTTG	GCCTATCCGTCTCGTCCGT
TLR7	141 bp	GTTCTATGGAGAGCCGGTGATA	ATTCTTTAGATTTGGCGGCATA
Ccl3	152 bp	CACTGCCCTTGCTGTTCTTC	GGCTGCTGGTTTCAAAATAGTC
Ccl4	162 bp	TCTCTCTCCTCTTGCTCGTGG	CTGGCTTGGAGCAAAGACTG
Bid	189 bp	CAGACCTGCTGGTGTTCGG	CCTGACTTTCAGAATCTGGCTC
Bcl2	154 bp	CTGAGAGAGGCAGGCGATG	CGATGCGACCCCAGTTTAC
C5a	158 bp	CATTGCTCCTCACCATTCCA	CAGAGGCAACACAAAACCCA
Egr2	154 bp	GCCCCTTTGACCAGATGAAC	GAGAATTTGCCCATGTAAGTGAA
Fos	157 bp	TGACAGATACACTCCAAGCGG	GGGAAGCCAAGGTCATCG
Junb	182 bp	AGTTACTCCCCAGCCTCTGC	GGTACGGTCTGCGGTTCCT
Map3k5	192 bp	GCCTAAACTAAAGTGGGAACACAT	CGAAGAACTTTATTGACCGCA
β-actin	263 bp	GAGACCTTCAACACCCCAGC	ATGTCACGCACGATTTCCC

### Histological assessment

Liver tissue sections (paraffin embedded) were stained with hematoxylin-eosin using a standard protocol, and analyzed by light microscopy under 400-fold magnification. Morphological criteria including vacuolization, swollen cytoplasm with disrupted cell and organelle membranes, and lytic nuclear changes served to determine necrosis. Hepatocyte apoptosis was assessed by the TUNEL assay kit (Roche, Switzerland). The protein expression of TNFα and iNOS in liver tissue was detected by immunohistochemistry; and the percentage of positive cells and the positive staining intensity were checked and scored using the Axiotis score standard by observation of 5 randomly chosen fields at 400-fold magnification [12]. The primary anti-TNFα and anti-iNOS antibodies were purchased from Cell Signaling Technology (USA). In this study, the liver histology was assessed by a single pathologist, unaware of the laboratory data.

### Statistical analysis

All animal experiments were done in triplicate, and the results were expressed as mean ± SD. Statistical comparisons were made by 1-way analysis of variance using SPSS 17.0 (SPSS Inc, Chicago, IL). The differences were considered statistically significant if *P* value less than 0.05.

## Results

### High-throughput sequencing data and DEGs in FHF mouse liver

**Figure 1:**
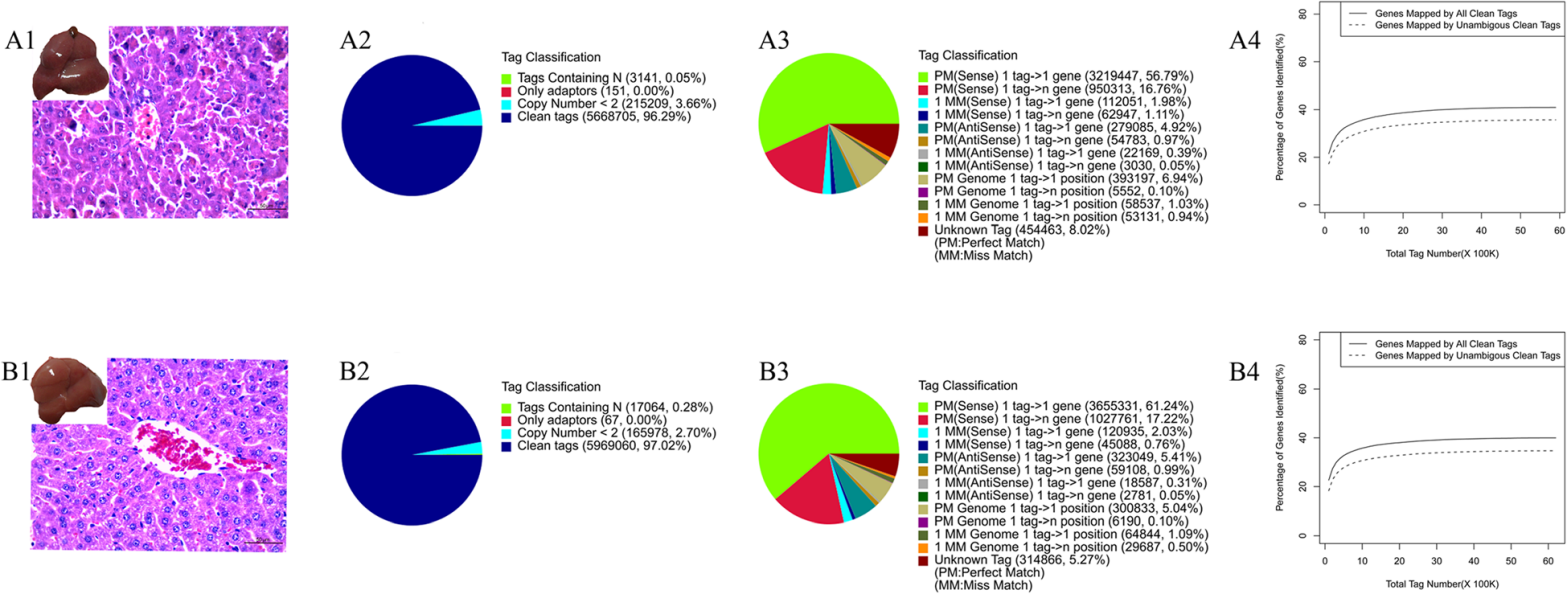
**Sequencing and mapping messages of hepatic mRNA profiling of normal and FHF mouse liver [A: fulminant hepatic failure (FHF) group, B: Normal control (NC) group]. (A1 and B1)** The mouse liver samples used to prepare the high-throughput sequencing libraries. **(A2 and B2)** The distribution of total tags in two libraries. **(A3 and B3)** The mapping of total clean tags in two libraries. **(A4 and B4)** Accumulation the genes mapped by all clean tags(solid line) and unique clean tags (broken line) in two libraries.

To obtain an overview of the gene expression profile in the development of fulminant hepatic failure, cDNA samples were prepared for FHF mouse model (6 h after the intraperitoneal injections of GalN/LPS, Figure 1-A1) and normal control mouse (Figure 1-B1), and then sequenced by using the Illumina sequencing platform. We obtained over 6.15 million and 5.88 million sequenced tags from normal and FHF liver, respectively. After filtering the adaptor sequences and removing the low-quality tags (including the tag with less than two copy), about 5.97 million (Figure 1-B2) and 5.67 million (Figure 1-A2) clean tags were left in the two libraries and used for further analysis, respectively; and there were about 4.12 million unique tags(68.99% of clean tags, Figure 1-B3) in normal control and 3.63 million unique tags (64.08% of clean tags, Figure 1-A3) in FHF model could be mapped to annotated mouse genes. Finally, 12611 (35.37% of reference genes, Figure 1-B4) and 13167 (35.93% of reference genes, Figure 1-A4) unique genes were detected and quantified from normal and FHF liver samples, respectively.

**Figure 2:**
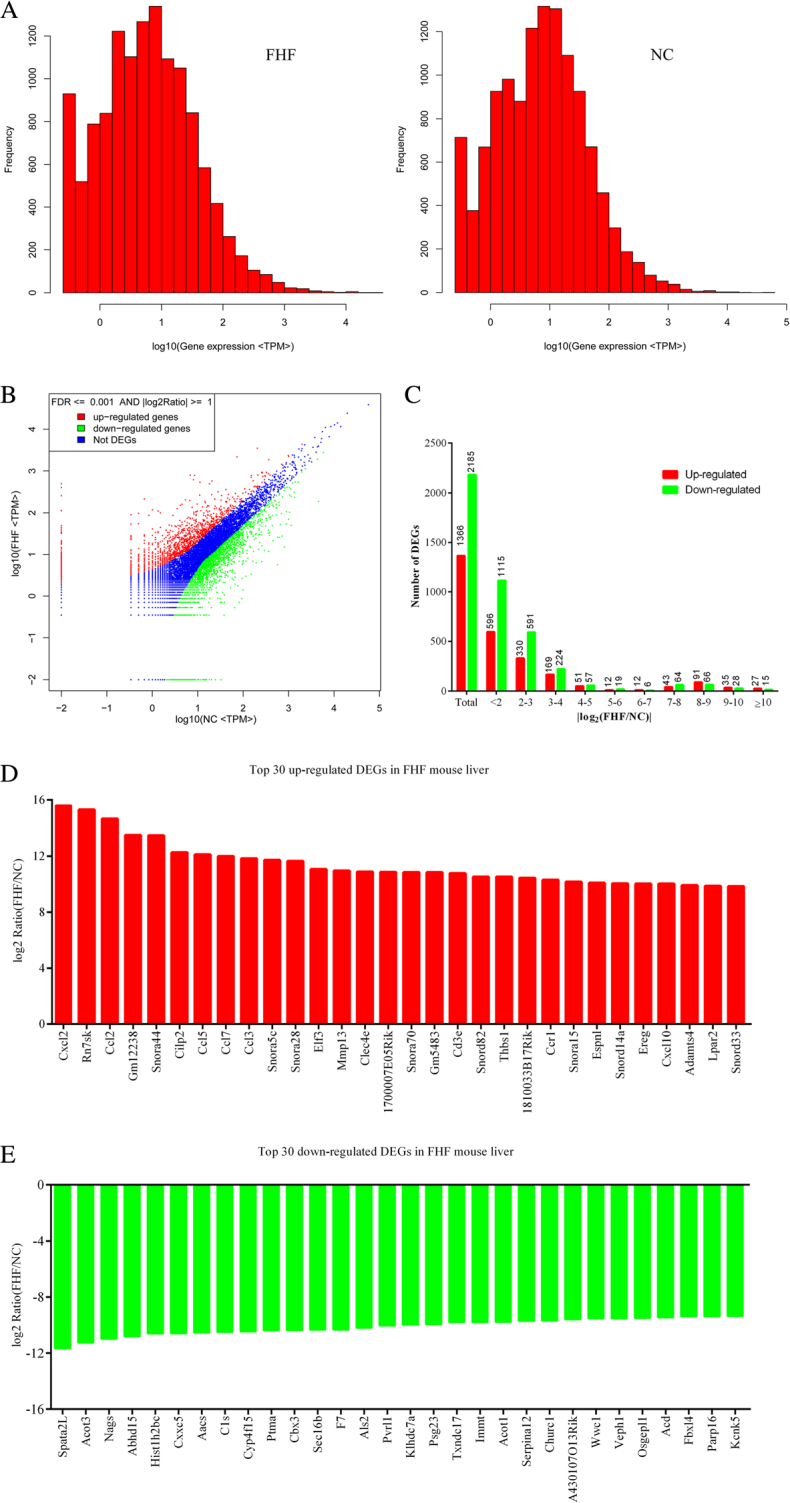
**The distribution of differentially expressed genes in FHF library. (A)** Distribution of gene expression between FHF (left) and NC groups (right). **(B)** Comparison of gene expression between FHF and NC libraries. **(C)** Distribution of DEGs by fold changes. **(D)** The top 30 up-regulated DEGs in FHF mouse liver. **(E)** The top 30 down-regulated in FHF mouse liver.

The distribution of gene expression between FHF and normal control mouse were presented in Figure 2A. The number of DEGs was quantified among above unique genes, and the absolute fold change no less than 1 and FDR less than 0.001 were used to define the DEGs. According to this definition, totally 3551 genes (including 1366 up-regulated and 2185 down-regulated) were differentially expressed between normal and FHF mouse liver sample (Figure 2B and C). In this study, Cxcl2 (chemokine [C-X-C motif] ligand 2), Rn7sk (RNA, 7SK, nuclear), Ccl2 (chemokine [C-C motif] ligand 2), Gm12238(predicted gene 12238), Snora44 (small nucleolar RNA, H/ACA box 44), Cilp2 (cartilage intermediate layer protein 2), Ccl5, Ccl7, Ccl3 and Snora5c (small nucleolar RNA, H/ACA box 5C) were in the top 10 fold change genes up-regulated in FHF mouse liver (Figure 2D), while Spata2L (spermatogenesis associated 2-like), Acot3 (acyl-CoA thioesterase 3), Nags(N-acetylglutamate synthase), Abhd15 (abhydrolase domain containing 15), Hist1 (histone cluster 1), Cxxc5 (CXXC finger 5), Aacs (acetoacetyl-CoA synthetase), C1s (complement component 1), Cyp4 (cytochrome P450, family 4), and Ptma(prothymosin alpha) were in the top 10 fold change genes down-regulated in FHF mouse liver (Figure 2E). The full list of DEGs was added as supplemental Table 1.

### The main biological processes and pathways altered in FHF mouse liver

**Figure 3:**
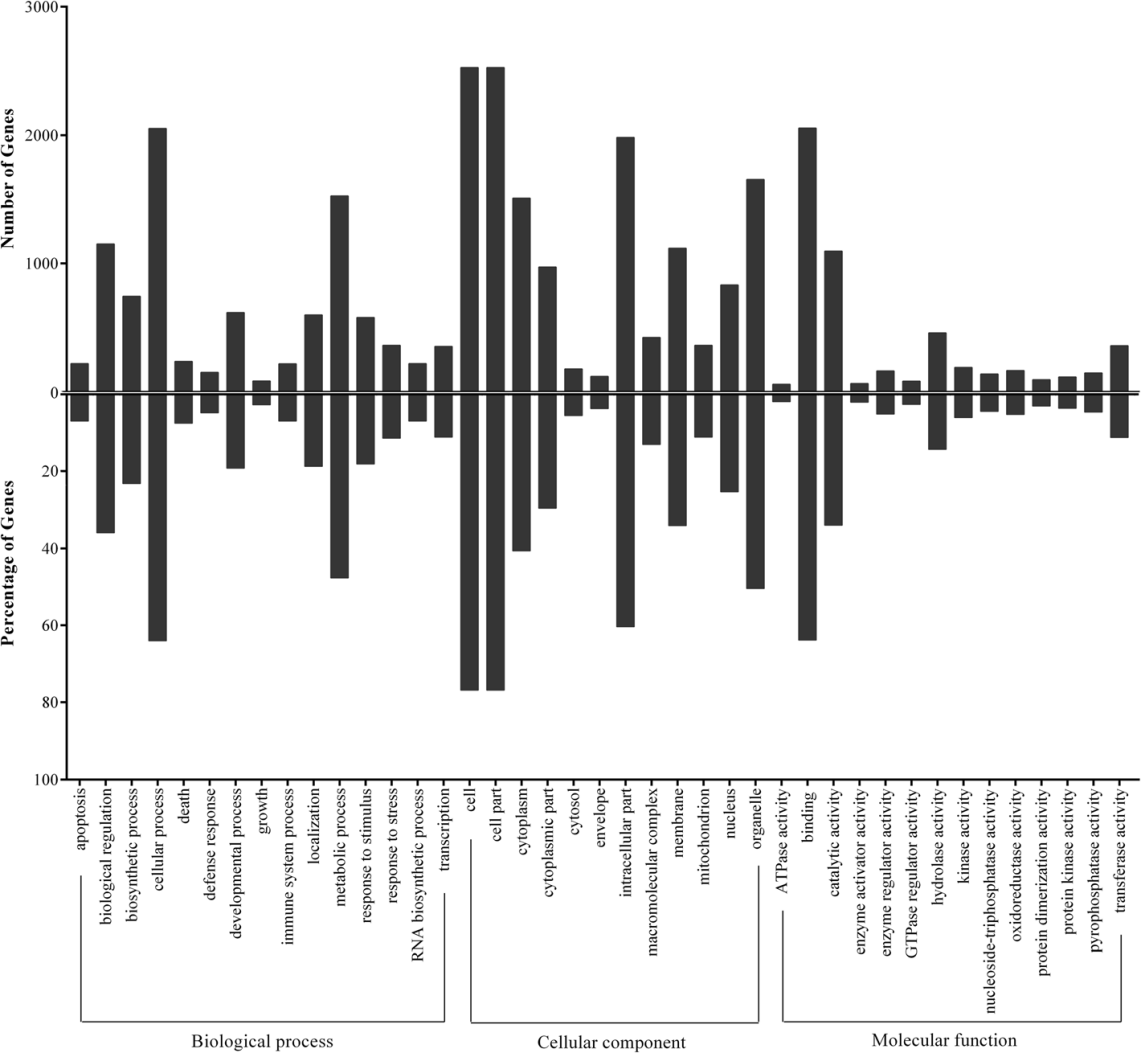
**Histogram presentation of Gene Ontology classification.** The results are summarized in three main categories: biological process, cellular component and molecular function. The upper half of y-axis indicates the number (up) and percentage (down) of genes in a category, and the lower half of y-axis indicates the percentage of a specific category of genes in that main category.

To investigate the possible biologic functions of the genes affected in FHF mouse liver, the above 1366 up-regulated and 2185 down-regulated DEGs were analyzed by Gene Ontology, and partial typical biological process, cellular componsent and molecular function were presented in Figure 3. Among biological process, DGEs were strongly enriched metabolic process, biosynthetic process, response to stimulus and response to stress, with 1527, 744, 579, and 365 genes, respectively.

Furthermore, KEGG pathway analysis was performed to further elucidate the biological functions of the gene clusters affected in FHF mouse liver, and a partial list of pathways significantly altered in FHF mouse liver was shown in Table 2, and following metabolic pathway (id: ko01100), peroxisome (id: ko04380), apoptosis (id: ko04210), adipocytokine signaling pathway (id: ko04920), chemokine signaling pathway (id: ko04062), and toll-like receptor signaling pathway(id: ko04620) were all involved.

**Table 2:** **A partial list of pathways significantly altered in FHF mouse liver**

**Pathway term**	**Pathway ID**	**DEGs tested**	***P*** **value**
Metabolic pathways	ko01100	396(15.03%)	2.22e-17
Peroxisome	ko04146	45(1.71%)	2.34e-07
ABC transporters	ko02010	24(0.91%)	9.89e-07
Protein processing in endoplasmic reticulum	ko04141	69(2.62%)	2.25e-06
Apoptosis	ko04210	39(1.48%)	1.59e-05
Drug metabolism - other enzymes	ko00983	28(1.06%)	1.98e-05
Chemokine signaling pathway	ko04062	76(2.89%)	4.80e-05
Adipocytokine signaling pathway	ko04920	37(1.40%)	0.0001
Toll-like receptor signaling pathway	ko04620	44(1.67%)	0.0001
Pantothenate and CoA biosynthesis	ko00770	10(0.38%)	0.0002
Fructose and mannose metabolism	ko00051	21(0.80%)	0.0003
Ascorbate and aldarate metabolism	ko00053	14(0.53%)	0.0003
Steroid biosynthesis	ko00100	12(0.46%)	0.0003

### The DEGs regulated by nuclear factor κB in FHF mouse liver

**Figure 4:**
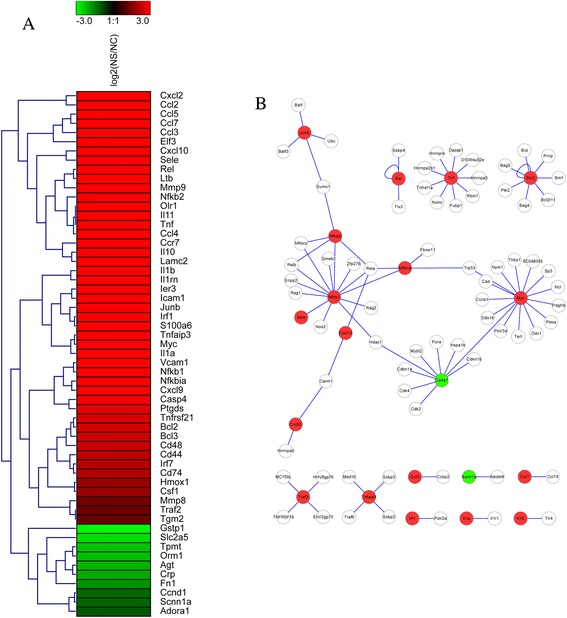
**The expression of DEGs regulated by NF-κB and their direct interaction in FHF library. (A)** Cluster image of expression patterns of DEGs that regulated by NF-κB. **(B)** Protein-protein direct interaction networks of NF-κB regulated DEGs.

In FHF, the hepatocytes were severely damaged and ordinary liver regeneration was impaired (Figure 1-A1), but the exact mechanism had not been fully elucidated. Since nuclear factor κB (NF-κB) family of transcription factors governed the cellular reaction to a variety of extracellular signals, which activated genes involved in inflammation, cell survival, cell cycle, immune cell homeostasis, etc. In this study, we focused our attention on the cytokines/signaling pathways and proteins that could be modulated by NF-κB, and about 57 DEGs modulated by NF-κB activation were found to be differentially expressed in the liver of FHF, including 47 up-regulated genes and 10 down-regulated genes (Figure 4A); and the direct interaction networks of those DEGs coding proteins were also presented in Figure 4B. For example, the widely concerned chemokines and its ligand in FHF (such as Cxcl 2, Ccl 2, Ccl 5, and et al) were significant observed in NF-κB pathway analysis.

**Figure 5:**
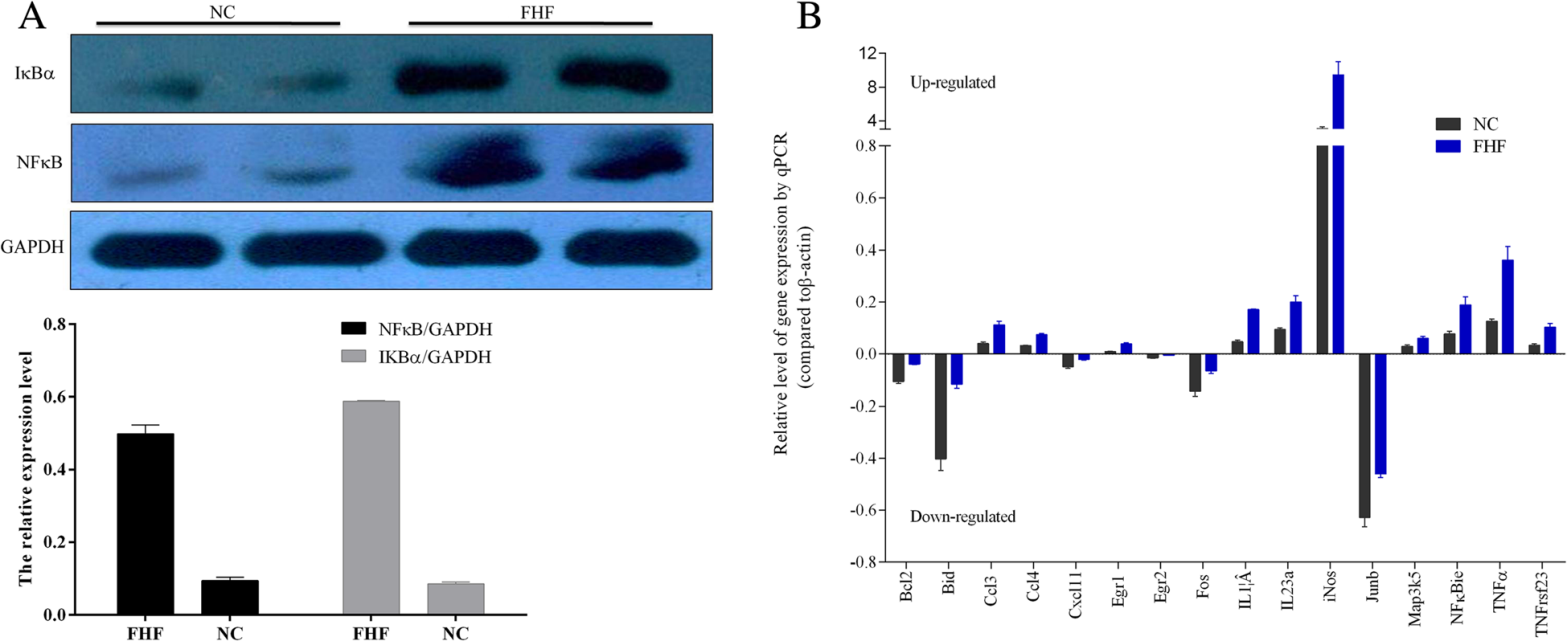
**Validations of NF-κB and its regulated DEGs in FHF liver samples. (A)** The expression of NF-κB and its negative regulator IκBα by Western blotting. **(B)** The mRNA levels of partial NF-κB regulated DEGs by qPCR.

In this study, the mRNA and protein expression of partial above-mentioned DEGs were also verified in FHF mouse liver. And the level of NF-κBp65 protein in FHF mouse liver were significantly elevated, about 5 times, compared with that in the healthy control mouse (0.499 ± 0.034 *vs.*0.095 ± 0.013, *P* = 0.004) (Figure 5A). Interesting, the expression of IκBα protein (0.588 ± 0.002 *vs.* 0.086 ± 0.008, *P* = 0.000), a negative feedback regulate of NF-κB, was also significantly increased about seven times in liver tissues of mice with FHF (Figure 5A). The mRNA levels of relevant DEGs modulated by NF-κBp65 were analyzed by quantitative PCR. As compared to normal control mouse, the levels of NFκBie (0.189 ± 0.054 *vs.* 0.078 ± 0.169, *P* = 0.027), TNFα (0.361 ± 0.093 *vs.* 0.126 ± 0.015, *P* = 0.012), IL1β (0.171 ± 0.003 *vs.* 0.047 ± 0.011, *P* = 0.000), iNOS (9.439 ± 2.712 *vs.* 3.083 ± 0.391, *P* = 0.016), CCL3 (0.112 ± 0.025 *vs.* 0.040 ± 0.01, *P* = 0.009) and CCL4 (0.074 ± 0.008 *vs.* 0.032 ± 0.002, *P* = 0.001) genes were significantly increased in hepatic tissues of FHF, and the levels of Bid (0.117 ± 0.027 *vs.* 0.403 ± 0.078, *P* = 0.004), Bcl2(0.039 ± 0.004 vs. 0.106 ± 0.013, *P* = 0.001) and Jub (0.46 ± 0.024 vs. 0.628 ± 0.062, *P* = 0.012) genes were decreased in hepatic tissues of FHF(Figure 5B). The above findings suggested that the significantly increased NF-κB protein positively may affect the balance of various cytokines in liver tissue, and participate in the aggravation of endotoxin-induced liver injury.

### Pre-block of NF-κB significant alleviates hepatic pathological damage and mortality of FHF mouse

**Figure 6:**
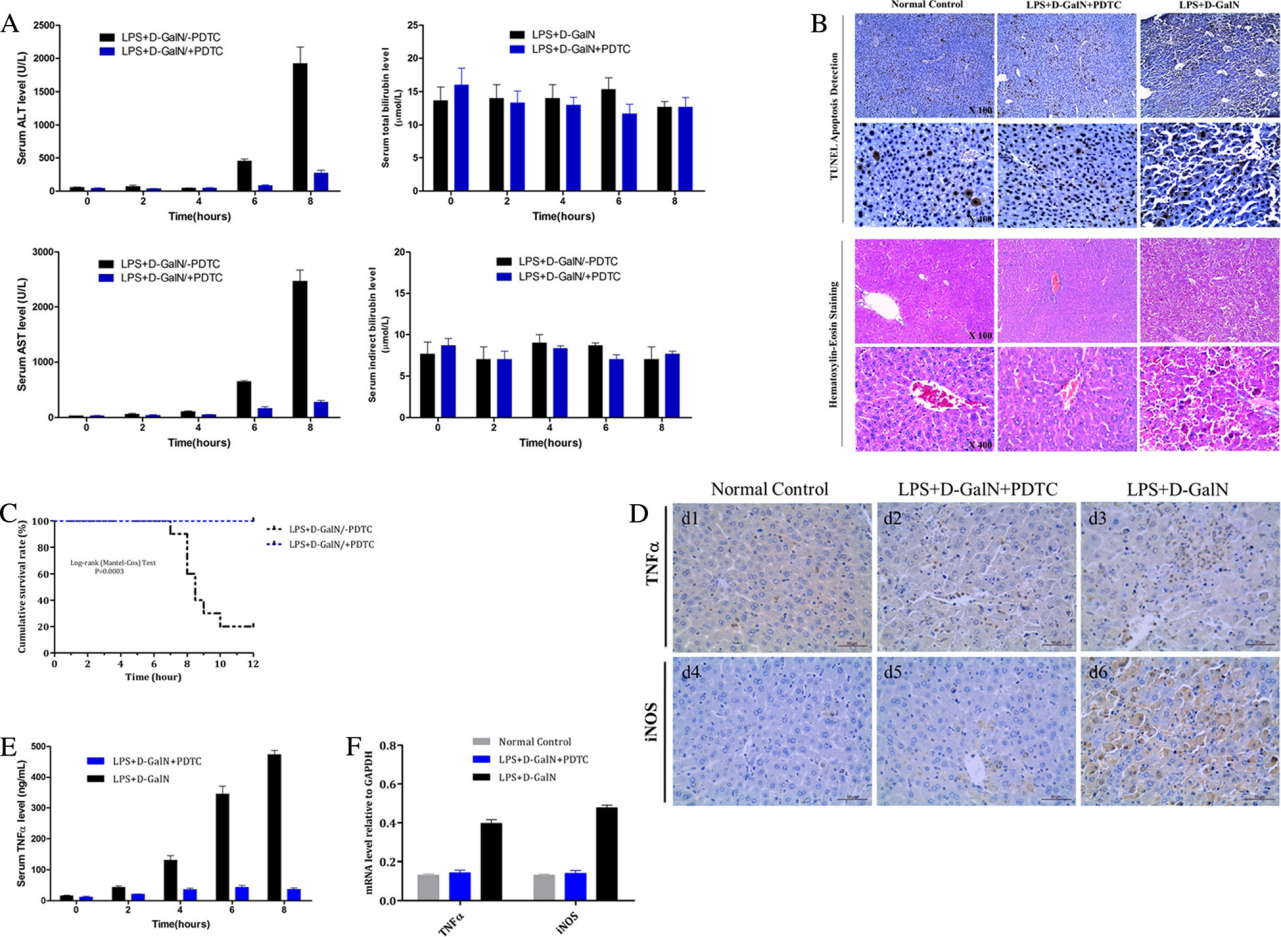
**Early administration of PDTC in alleviating hepatic pathological damage and preventing FHF occurrence. (A)** The biochemical parameters of liver function. **(B)** TUNEL assay H&E and staining results. **(C)** 12-hour cumulative survival rate. **(D)** The expression of TNFα and iNOS detected by immunohistochemistry assay. **(E)** The serum protein levels of TNFα detected by ELISA. **(F)** The mRNA levels of TNFα and iNOS by qPCR.

To clarify whether increased NF-κB protein participate in the occurrence and development of FHF, the expression of NF-κB was pre-blocked by chemical inhibitor PDTC, 12 h before the construction of experimental FHF mouse model. As shown in Figure 6, NF-κB inhibition resulted in remarkable improvement of biochemical parameters of liver function, which was demonstrated by the lower levels of serum ALT, AST, TBil and IBil in FHF as compared to those in normal control mouse (Figure 6A). And H&E staining and TUNEL assay results revealed that the hepatic apoptosis and necrosis was strongly suppressed in FHF after NF-κB was pre-blocked by PDTC (Figure 6B). Importantly, the cumulative 12-month survival rates of FHF mice model was significantly increased from 20% to 100% when NF-κB was pre-blocked (Figure 6C).

In immunohistochemistry assay, the number of positive cells and intensity of the reaction of TNFα and iNOS were obvious in liver tissue of FHF (Figure 6D); and after NF-κB was pre-blocked, the expression of TNFα and iNOS in liver tissue were both significantly decreased (Figure 6D,E and F). In summary, our data suggested that suppression of NF-κB activation or expression mediated by chemical inhibitor PDTC could significantly improve liver function and reduce mortality of FHF mouse model, and NF-κB would be a potential new therapeutic target for FHF.

## Discussion

At present, the exact mechanisms of the pathophysiological events in the liver leading to FHF are not fully understood, there is still no available particularly effective therapy except for liver transplantation; however, the latter is also limited by a severe shortage of donor livers [13]. Thus, there is an urgent need to explore new effective treatment measures. In past decade, some host genes or their encoded moleculars (including cytokines and chemokines) had been discovered that were associated with hepatocyte unnatural death and participated in the occurrence of FHF [14]. However, the interactions of vast majority of cytokines/chemokines with each other remained unclear in the process of FHF.

Currently, detecting changes in gene expression of host and their interaction networks have become an increasingly important topic in FHF, and the genes differentially expressed in FHF are of foremost interest to both basic and clinical researches [15]. With the assistance of the new-generation high throughput sequencing technology, DGE has be employed to economically and quickly capture the whole genome of the gene expression of certain tissue of a species under specific conditions [16]. In this study, this technology was firstly used to identify DEGs of mouse liver tissue under different external stimuli conditions, and it was also the first reported attempt to reveal the global network of DEGs in FHF. It is worth to mention that our results have provided a variety of potential candidate targets for pathogenic mechanism and new drug development of FHF, and all raw data could be obtained in Additional file 1: Table S1.

Though hepatotropic virus infection is the main cause of FHF in clinical practice, there is still no hepatotropic virus induced FHF animal model available at present, which greatly limits the investigations of related molecular mechanisms and new treatment strategies of FHF. As we know, the activation of hepatic macrophages and its-associated inflammation are extremely common in patients with FHF, and this situation is largely related to endotoxin exposure [17]. So, most scholars had to choose endotoxin-induced animal model to conduct related research work on FHF in past years, though it has defects in reflecting the role of hepatotropic virus in the pathogenesis of massive hepatic necrosis. Based on this present situation, the D-GalN/LPS-induced acute liver damage mouse model has been used to yield the global gene expression profile of FHF in present study; and a total of 3551 DEGs were successfully detected in liver tissue of FHF, including 1366 up-regulated and 2185 down-regulated DEGs. And majority of those detected DGEs were strongly enriched metabolic process, biosynthetic process, response to stimulus and response to stress. As we know, liver is a multifunctional organ involved in important metabolic functions, synthesis of plasma proteins and storage of lipids and retinol, and the failure of liver function could result in serious metabolic disorder of sugar, fat and protein. Thus the highly enriched metabolic process observed in this study was consistent with the objective fact. Although we did not conduct in-depth verification of the relevant DEGs on liver cellular metabolism, the global interaction map of corresponding DEGs reported in this study would provide important clues to future studies.

Excessive apoptosis and abnormal necrosis are prominent features of FHF [18]. According to analysis of biological process, high to 579 and 365 DEGs involved in response to stimulus and response to stress, respectively. To shed more lights into the function roles of genes responsible for the progression of FHF, we further investigated the hepatocyte damage associated apoptosis and chemokine signaling pathways by the enrichment analysis of DEGs among normal and FHF liver samples, and found there were 39 DEGs in apoptosis pathway and 76 DEGs in chemokine signaling pathways. From the large number of DEGs in above two-mentioned pathways, it’s easy to see that inflammatory processes play a fundamental role in liver tissue damage, and the complexity of inflammatory cytokine network involved in the occurrence and development of FHF. In recent years, many in vitro and in vivo studies have examined the contribution of components of the NF-κB signaling pathways to the regulation of liver cell damage and repair, in particular, of liver cell death and regeneration in liver damage development [19,20]. However, the exact role of NF-κB in FHF in vivo is still controversial [21]. In this study, according to the clues provided by DGEP experiment, we performed a cluster analysis of DEGs in NF-κB pathway, and there were 32 DEGs up-regulated or down-regulated in FHF. Additionally, the expression levels of NF-κBp65 and IκBα proteins were both increased significantly in liver tissue. So, we inferred that the activation of NF-κB mediated signaling pathways played important roles in the development of FHF. As the gene transcription of IκBα was regulated by NF-κB, the high expression of IκBα proteins in FHF could be explained by the highly activated NF-κBp65. In addition, the high expression of IκBα proteins in this experiment may also execute a negative feedback regulation of NF-κB overexpression in the development of FHF [22].

In this study, after the activation approach of NF-κB were pre-blocked, we found that D-GalN/ LPS intraperitoneal injection could not induce significantly liver damage and all mouse survived. So we believed that the overexpression of NF-κB or highly activated NF-κB should participate in the progression of liver damage to FHF.

Though the higher expression levels of NF-κB have been reported to protect cells from apoptosis and necrosis in some vitro experiments [23], the overexpression of NF-κB also would activate and enhance the host non-specific and specific immune response, and therefore result in tissue injury and organ dysfunction [24]. As we know, the biological function of certain molecules would be affected by the changes of surrounding environment of cells, so the vast difference between in vitro and in vivo environment is likely to influence the functions of NF-κB. So, we believe that the overexpression of NF-κB should mediate or aggravate the liver cell necrosis in the process of FHF. Considering that oxidative stress also has been shown to aggravate the hepatotoxicity induced by LPS/D-GalN and PDTC is also an antioxidant, BAY-11-7082 (another inhibitor of NF-κB) also was used to verify the function of NF-κB, and the result was in good agreement with PDTC experiment (data unshown).

Previous studies showed NF-κB was able to bind promoter and enhancer regions containing κB sites, and TNFα, iNOS, IL-2, IL-6, IL-8, GM-CSF, ICAM-1, MHC-1, TNFβ, Toll -like receptors, chemokines and chemokine receptor (CR) were all regulated by NF-κB [25-27]. So in this study, the high-level expression of cytokines (such as TNFα and iNOS) and chemokines were associated with the over-activated NF-κB complex, and their direct and indirect damaging effects on liver cells also partially contributed to the pathogenic role of NF-κB activation [28].

## Conclusions

This study is the first genome-wide effort to investigate the transcriptional changes in FHF development. Though it is impossible to discuss the relevance and impact of all genes and clusters that were differentially expressed in this study, present study still demonstrates the possibility of NF-κB gene as a potential therapeutic target for FHF.

### Ethical approval

All animal experimental procedures were approved by the Institutional Animal Care and Use Committee of Sichuan University.

## Additional file

Additional file 1: Table S1. The full list of DEGs in FHF mouse liver.
